# Extent and consistency of linkage disequilibrium and identification of DNA markers for production and egg quality traits in commercial layer chicken populations

**DOI:** 10.1186/1471-2164-10-S2-S2

**Published:** 2009-07-14

**Authors:** Behnam Abasht, Erin Sandford, Jesus Arango, Petek Settar, Janet E Fulton, Neil P O'Sullivan, Abebe Hassen, David Habier, Rohan L Fernando, Jack CM Dekkers, Susan J Lamont

**Affiliations:** 1 Department of Animal Science and Center for Integrated Animal Genomics, Iowa State University, Ames, IA 50011, USA; 2 Hy-Line International, Dallas Center, IA 50063, USA

## Abstract

**Background:**

The genome sequence and a high-density SNP map are now available for the chicken and can be used to identify genetic markers for use in marker-assisted selection (MAS). Effective MAS requires high linkage disequilibrium (LD) between markers and quantitative trait loci (QTL), and sustained marker-QTL LD over generations. This study used data from a 3,000 SNP panel to assess the level and consistency of LD between single nucleotide polymorphisms (SNPs) over consecutive years in two egg-layer chicken lines, and analyzed one line by two methods (SNP-wise association and genome-wise Bayesian analysis) to identify markers associated with egg-quality and egg-production phenotypes.

**Results:**

The LD between markers pairs was high at short distances (r^2 ^> 0.2 at < 2 Mb) and remained high after one generation (correlations of 0.80 to 0.92 at < 5 Mb) in both lines. Single- and 3-SNP regression analyses using a mixed model with SNP as fixed effect resulted in 159 and 76 significant tests (P < 0.01), respectively, across 12 traits. A Bayesian analysis called BayesB, that fits all SNPs simultaneously as random effects and uses model averaging procedures, identified 33 SNPs that were included in the model >20% of the time (*φ *> 0.2) and an additional ten 3-SNP windows that had a sum of *φ *greater than 0.35. Generally, SNPs included in the Bayesian model also had a small *P*-value in the 1-SNP analyses.

**Conclusion:**

High LD correlations between markers at short distances across two generations indicate that such markers will retain high LD with linked QTL and be effective for MAS. The different association analysis methods used provided consistent results. Multiple single SNPs and 3-SNP windows were significantly associated with egg-related traits, providing genomic positions of QTL that can be useful for both MAS and to identify causal mutations.

## Background

Genetic variation within breeds or lines is the primary source for genetic improvement in livestock. Large rates of genetic improvement have been achieved in poultry by selecting breeders with high breeding values based on phenotypic information. Following advances in molecular genetic technologies and availability of DNA markers, interest has rapidly grown in identifying quantitative trait loci (QTL) that genetically control important traits for application in marker-assisted selection (MAS) programs [1,2]. Various experimental designs have been used to date to identify QTL for economically important traits in chickens [3]. Although these studies have successfully identified many (about 700) QTL, the direct application of these results in commercial lines has been limited [4]. Identification of 2.8 million single nucleotide polymorphisms (SNP) in the chicken genome, continued advances in sequencing and high-throughput genotyping methods, and progress in developing computational methods for analyzing high-density SNP data [5-8], have markedly improved the feasibility of using genomic information in poultry breeding.

Understanding the linkage disequilibrium (LD) that exists in a population is necessary for effective application of MAS. Linkage disequilibrium measures the non-random association of alleles at two or more loci in the population. Within a closed breeding population, the extent of LD is affected by the distance between the loci and historical effective population size [9]. In general, the shorter the distance between two loci and the smaller the effective population size, the greater their LD is expected to be. Because of years of selective breeding, farmed animals tend to have lower effective population sizes and, therefore, greater LD than most human populations. The density of markers needed for QTL identification is determined by the extent of LD within a population. Effective MAS also requires sustained LD between markers and QTL over generations. Andreescu et al. [10] examined the extent and consistency of LD across nine commercial broiler breeding lines, but did not evaluate consistency of LD over generations. The latter was evaluated by Heifetz et al. [11] but based on a limited number of microsatellite markers. The first objective of the current study, therefore, was to assess the level and consistency of LD between SNPs over consecutive years in two elite layer chicken breeding lines.

A variety of statistical methods can be used for analysis of high-density SNP data to identify QTL regions or SNPs associated with phenotypic traits using LD in closed breeding populations. These methods were reviewed by Dekkers et al. [12] and some were compared by simulation by Zhao et al. [13], including single SNP analyses and haplotype analysis methods, which fit each SNP or SNP window separately, as well as genomic selection approaches, which fit all SNPs across the genome simultaneously [6]. These will henceforth be referred to as SNP-wise and genome-wise models. For the genome-wise models, Meuwissen et al. [6] showed that genetic value can be predicted with higher accuracy using Bayesian methods that estimate a variance associated with each marker (or haplotype), compared to a method which assumes equal variance associated with each marker. Among two Bayesian methods called BayesA and BayesB, the latter had higher accuracy in predicting genetic values. In contrast to BayesA, the BayesB method uses a prior that assumes that many marker loci have no association with phenotype and uses model averaging to estimate effects associated with each marker. Although initially developed to predict breeding values using high-density SNP data, these genome-wise models can also be used to identify SNPs that are associated with QTL, as demonstrated by Xu [7]. Thus, the second objective of this study was to use and compare SNP-wise and genome-wise approaches to identify markers associated with production traits in one elite egg-layer breeding line.

## Methods

Data from two elite breeding lines of egg-laying chickens from Hy-Line Int., Line 1 (White Egg) and Line 2 (Brown Egg), were analyzed. The data included average adjusted progeny performance of sires for two egg-production and 10 egg-quality traits, pedigree information, and genotypes for a 3,000 SNP panel. Phenotypic data for each sire were the average performance of his daughters, with each daughter's phenotype adjusted for random effects of half of the Estimated Breeding Value (EBV) of the daughter's dam and for the fixed effect of hatch, as estimated by routine genetic evaluation procedures utilized by Hy-Line, International. Traits were classified into two categories based on age of measurement: early (first 3 egg weight, or measured at week 26) and late traits (measured at week 38). Early traits included Early Albumen Height (EAH, mm), First 3 Egg Weight (E3, gr), Early Egg Weight (EEW, gr), Early Production (EPD, % egg production), Early Shell Quality (EPS, gr in pin point pressure), Sexual Maturity (SM, day) and Early Yolk Weight (EYW, gr). Late traits included Late Albumen Height (LAH, mm), Late Egg Weight (LEW, gr), Late Production (LPD, % egg production), Late Shell Quality (LPS, gr in pin point pressure) and Late Yolk Weight (LYW, gr).

The DNA samples from sires in two consecutive generations (2003 and 2004) and from their paternal ancestors (3 to 6 generations back) were genotyped for about 3000 SNPs using an Illumina SNP array [14] that has been previously described [10,15]. In total, 132 and 131 sires were genotyped in Line 1 and Line 2, respectively.

### Linkage disequilibrium analyses

Linkage disequilibrium was analyzed using the SNP data for chromosomes 1 and 4 on roosters from the two lines from two consecutive years (2003 and 2004), reflecting two subsequent generations. These two chromosomes were chosen because they had sufficiently larger numbers of SNPs to obtain accurate results. In addition, results are expected to be representative of other chromosomes (see discussion section). The numbers of genotyped roosters were 60 and 67 for Line 1 and 2, respectively, in year 2003; and 42 and 20 in year 2004. Chromosomes 1 and 4 had 449 and 184 genotyped SNPs, which were spaced on average 450–500 kb apart. Because low minor allele frequency (MAF) can skew measures of LD [10], only SNPs with MAF greater than or equal to 0.05 were used for LD analyses.

The SNP data were analyzed using Haploview [16] and PowerMarker [17] to measure the LD between all pairs of markers by r^2 ^and r. To assess the decline of LD with distance, r^2 ^was plotted against distance and a moving average of LD was calculated. A predicted LD curve was estimated by fitting the Sved [9] equation to the r^2 ^data: r^2 ^= 1/(1+4*N_e _*d), where d is distance in Morgans estimated using 2.4 cM/Mb for both chromosomes, and N_e _is the effective population size. This model was fitted to data from each line and each chromosome using methods described by Zhao et al. [18]. To assess consistency of LD across generations and between the two lines, correlations of LD were calculated for matching marker pairs, by distance between markers.

### SNP association analyses

For the purpose of detecting SNPs associated with traits, which was performed in line 1 only, Illumina's top A and B allele calls [19] for SNPs that were segregating in the population (MAF>0) were coded as 0 or 1, respectively, and the number of copies of the 0 allele that a genotyped sire carried at each locus (0, 1 or 2) was included in statistical models. For missing genotypes (<1% of all genotype calls), twice the frequency of allele 0 among genotyped sires within the line was used as the expected number of 0 alleles for that individual.

Initial analyses of SNP data of Line 1 showed 22 roosters with genotypes for one or more SNPs that were incompatible with the genotype of the sire (eg., genotype = 0 and sire genotype = 2). For most of these individuals, incompatibility was limited to one SNP, which was considered as a genotyping error (<0.005% of total genotypes) and coded as missing. For individuals with multiple incompatible SNPs (n = 21 to 60), parentage testing was carried out to determine the most likely sire. Briefly, SNP genotypes of these individuals were compared with the SNP genotypes of all genotyped individuals and the number of incompatible SNP genotypes was counted for each pair of individuals. For each of these individuals, there was a single sire in the previous generation with fully compatible SNP genotypes and these were, therefore, used as their sire in the analysis.

Association analyses were conducted using two types of analyses: 1) SNP-wise analyses, in which each SNP or each window of three consecutive SNPs was fitted separately, along with a polygenic effect, and 2) Genome-wise analyses, in which all SNPs were fitted simultaneously, using the BayesB method of Meuwissen et al. [6].

### SNP-wise analyses

Two different mixed models were used for the SNP-wise analyses: a 1-SNP model and a 3-SNP model. Both models were implemented using the Mixed Model procedure of SAS based on programs developed by Hassen et al. [20] and using SAS macro LORG [21] to fit a polygenic effect with relationships. The general model equation used was:

**Y **is an n × 1 vector of average adjusted daughters' performance for n sires

**X**_gen _is design matrix which relates observations to fixed generation effects

**Gen **is a vector of solutions for the generation effect

**X**_i _is the design matrix for SNP effects, with elements equal to the number of 0 alleles carried by each sire. For the 1-SNP analysis, this is an n × 1 matrix with elements equal to the number of 0 alleles carried for SNP i. For the 3-SNP analysis, this is an n × 3 matrix with elements corresponding to the number of 0 alleles for SNPs i-1, i, and i+1.

***β***_i _is a 1 × 1 (1-SNP analysis) or a 3 × 1 (3-SNP analysis) vector of SNP allele substitution effects, fitted as fixed effects.

**Z **is an incidence matrix relating random sire effects to the vector of observations

**s **is a vector of random effects of sires, assumed distributed normal with mean 0 and variance **A**, where **A **is the additive genetic relationship matrix [22] derived from four generations of pedigree of male and female ancestors, and  is the sire variance

**e **is a vector of random error effects, which was assumed distributed normal with mean 0 and variance **D** where **D **is a diagonal matrix with elements equal to the reciprocal of the number of progeny included into each sire's progeny average, and  is the error variance.

Estimates of  and  were obtained by ASReml [23] using the above model but without SNP effects.

The significance of SNP effects was obtained from a likelihood ratio test of the full model that included SNP effects to a reduced model without SNP effects [22]. Associated *P*-values were obtained from a chi-square distribution with one (for the 1-SNP model) or three (for the 3-SNP model) degrees of freedom.

### Genome-wise analyses

The BayesB method of Meuwissen et al. [6] was used to identify SNPs associated with traits by fitting all SNPs across the genome simultaneously. In addition to the mean and random markers and error effects that were modelled in the BayesB method of Meuwissen et al. [6], a polygenic effect of sires was also included. The model fitted to the average adjusted daughters' performances of sires from the two last generations was:

where **Y**, **X**_i_, *β*_i_, **Z**, **s**, and **e **are as in the 1-SNP analysis model described previously, *μ *is an intercept, I_i _is a 0/1 indicator variable indicating whether SNP i is included in the model (see below), and the summation is over all SNPs that are segregating at MAF ≥ 0.05. Substitution effects *β*_i _were fitted as random with variance . The prior probability that a SNP has genetic variance greater than zero ( >0 and so I_i _= 1), 1-*π*, was set equal to 0.05 [6]. The prior distribution of the variance of such loci, , was an inverted chi-square distribution *X*^-2^(*v*, *S*), with parameters *v *= 4.2 [6] and , where  is the expected additive genetic variance, which was set to 4, where  is the estimate of the sire variance obtained from the ASReml analysis described previously, *k *is the expected number of segregating QTL, which was set equal to 200 and  is mean allele frequency variance, which was set equal to 0.5. Note that the *S *parameter used here differs from what was used by Meuwissen et al. [6] because here  was taken to be [24,25] rather than /*k*, as in Meuwissen et al. [6]. The Markov chain Monte Carlo (MCMC) algorithm used to fit this model was run for 150,000 iterations, which included running one Gibbs chain in each iteration for sampling *μ*, , **s**, , and *β*_i_, and running 100 cycles of the Metropolis-Hastings algorithm within each Gibbs chain for sampling . The first 100,000 cycles were removed from the analysis as burn-in. If, in an iteration of the MCMC,  = 0 for SNP i, then that SNP was not included in the model in that iteration (I_i _= 0).

Polygenic effects, **s**, and polygenic variance, , were sampled using a fully blocked implementation of the Gibbs sampler presented by García-Cortés and Sorensen [26] in the context of the Gaussian linear model. The prior distribution of polygenic effects **s **was assumed Normal (0, **A**), where **A **is the numerator relationship matrix, derived as described previously. The prior distribution of  was an inverted chi-square distribution *X*^-2^(*v*, *S*), with *v *= 4.2 and , where  is the expected polygenic variance which was set to half of the genetic variance estimated for the model without SNPs using ASReml.

The fraction of cycles with I_i _= 1 for each SNP gives a marginal posterior probability (*φ*_*i*_) of a SNP being associated with the trait, conditional on all other markers included in the model. An association was considered significant if *φ*_*i *_> 0.2. Given a prior probability of a SNP having non-zero variance of 1-*π *= 0.05, *φ*_*i *_> 0.2 can be interpreted as the probability that a SNP has non-zero variance at least 4 times more often than expected. However, this number is likely underestimated as effects of a QTL could be distributed across multiple correlated SNPs that may be genotyped in the QTL region (see discussion). Furthermore, if the sum of *φ*_*i *_for a window of 3 adjacent SNPs was greater than 0.35 (excluding windows already identified as significant by containing a SNP with *φ*_*i *_> 0.2), that 3-SNP window was considered to be significantly associated with the trait. The rationale for applying this approach is that the sum of *φ*_*i *_for 3 adjacent SNPs might represent an important signal from a region in which no single SNP was above the *φ*_*i *_> 0.2 threshold.

In addition to estimating trait polygenic and residual variances by ASReml, as described previously, they were also estimated using the Bayesian model without fitting SNP effects. In these analyses, the mean of the prior distribution of the polygenic variance was set equal to the estimate obtained from ASReml.

## Results

### Linkage disequilibrium analyses

**Figure 1:**
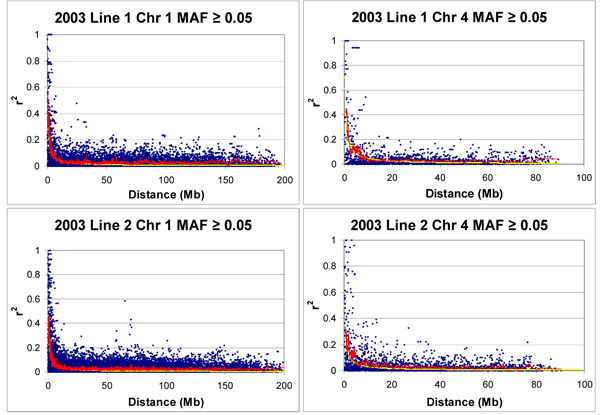
**Plots of LD measured by r^2 ^vs. distance in Megabases**. The red line is a moving average of the data and the yellow line is the predicted LD curve.

Plots of the estimated LD and distance between marker pairs displayed a sharp decline of LD with increasing distance (Figure 1). Lines showing the moving average of the measured LD and the predicted LD curve from fitting the Sved [9] equation followed each other well, indicating that the population behaved as predicted with respect to decline of LD over distance. Estimated effective population sizes tended to be less than 30, reflective of the history of stringent selection in closed populations for egg-laying chickens, but had large standard errors. Estimated effective population size was smaller for line 1 than line 2, which was also reflected in the number of segregating SNPs, which was greater for Line 2 than for Line 1.

The LD between pairs of markers that were segregating in both years (9,453 and 1,653 pairs for Line 1 for chromosomes 1 and 4, respectively, and 19,110 and 2,016 pairs for Line 2) was relatively consistent across years, as quantified by the correlation between LD (Tables 1 and 2). As expected, correlations tended to be greater for marker pairs that were closer, for both r and r^2^, and this pattern was consistent across both years and chromosomes.

**Table 1:** Correlation of LD measured by r^2 ^and r over selected Distances between Years, Chromosome 1^1^

	0–1 Mb	1–5 Mb	5–10 Mb	10–50 Mb	50–100 Mb	>100 Mb
r^2^						
Line 1	0.92	0.89	0.62	0.23	0.14	0.14
Line 2	0.88	0.73	0.35	0.08	0.06	0.12
r						
Line 1	0.95	0.87	0.73	0.32	0.21	0.29
Line 2	0.89	0.79	0.46	0.24	0.16	0.21

^1^All correlations were significantly difference from 0 (*P *< 0.05)

**Table 2:** Correlation of LD measured by r^2 ^or r over selected Distances between Years, Chromosome 4^1^

	0–5 Mb	5–15 Mb	15–25 Mb	25–50 Mb	> 50 Mb
r^2^					
Line 1	0.92	0.84	0.18	0.05 *	0.14
Line 2	0.89	0.15	0.09 *	0.01 *	-0.006 *
r					
Line 1	0.72	0.59	0.34	0.33	0.16
Line 2	0.76	0.46	0.22	0.10	0.08 *

^1^Correlations marked with an asterisk were not significantly difference from 0 (*P *> 0.05)

Fewer marker pairs were available to compute correlations of LD between lines, than between years within lines, due to differences in SNP fixation and frequencies between the lines; shared SNP pairs between lines on chromosomes 1 and 4 were 2,850 and 465 pairs, respectively, in 2003, and 2,556 and 378 pairs in 2004. Correlations of LD between lines tended to be close to zero, even at short distances (-0.11 to 0.23 for r^2^, -0.21 to 0.27 for r over all distances), indicating that there was little to no consistency of LD between the lines (Table 3 and 4). This was as expected, because the lines have no known genetic relationship; Line 1 was a white egg line and Line 2 was a brown egg line.

**Table 3:** Correlation of LD measured by r^2 ^or r over selected Distances between Lines, Chromosome 1^1^

	0–5 Mb	5–10 Mb	10–50 Mb	50–100 Mb	100–150 Mb	> 150 Mb
r^2^						
2003	0.24	0.06 *	0.03 *	0.01 *	-0.04 *	-0.02 *
2004	0.10 *	0.01 *	-0.03 *	-0.02 *	-0.03 *	0.07 *
r						
2003	-0.01 *	-0.01 *	0.002 *	0.01 *	-0.03 *	0.05 *
2004	0.003 *	-0.14 *	-0.03 *	0.00 *	-0.01 *	0.04 *

^1^Correlations marked with an asterisk were not significantly difference from 0 (*P *> 0.05)

**Table 4:** Correlation of LD measured by r^2 ^or r over selected Distances between Lines, Chromosome 4^1^

	0–5 Mb	5–15 Mb	15–25 Mb	25–50 Mb	> 50 Mb
r^2^					
2003	0.27	-0.12 *	0.20 *	-0.11 *	0.18
2004	0.15 *	-0.06 *	-0.11 *	0.06 *	-0.07 *
r					
2003	-0.21 *	0.03 *	0.15 *	0.01 *	0.06 *
2004	-0.06 *	0.08 *	0.05 *	0.17 *	0.09 *

^1^Correlations marked with an asterisk were not significantly difference from 0 (*P *> 0.05)

### SNP association analyses

Estimates of heritability for the traits, calculated from sire and residual variances components obtained by ASReml, varied from 0.05 to 0.84 (Table 5). The estimates of polygenic and residual variances using the Bayesian model without fitting SNP effects were on average 4% and 6% different, respectively, from those obtained by ASReml (Table 5). By fitting SNP effects in the genome-wise model, the polygenic variance decreased by 50%, while residual variance increased on average by 4% (averaged across all traits), compared to the model used without fitting SNP effect (Table 5).

**Table 5:** Estimates of sire polygenic and residual variances using the Bayesian genome-wise model, as a proportion of estimates obtained by ASReml without SNPs

	Genome-wise^1^				ASReml^2^
		
	with SNP effect		without SNP effect		
Trait^3^	Polygenic	Residual	Polygenic	Residual	h^2^
EAH	0.29	0.91	0.89	1.13	0.05
E3	0.51	1.05	1.01	0.90	0.13
EEW	0.60	1.33	0.97	1.13	0.44
EPD	0.52	1.00	1.06	0.97	0.05
EPS	0.65	1.14	0.97	1.04	0.08
SM	0.54	1.19	0.99	1.09	0.18
EYW	0.44	1.07	1.01	0.96	0.16
LAH	0.56	1.03	1.01	0.99	0.56
LEW	0.46	0.97	1.00	1.01	0.25
LPD	0.42	0.94	0.96	1.05	0.21
LPS	0.48	0.94	0.98	1.01	0.19
LYW	0.50	1.36	1.18	1.14	0.84

^1 ^Polygenic and residual variances for each trait were estimated using genome-wise model (MCMC method) with or without fitting SNP effect in the model and presented as proportional to the polygenic and residual variances estimated using ASReml.

^2 ^Polygenic and residual variances for each trait were estimated using ASReml and used for estimating heritability, .

^3 ^Trait abbreviations: Early Albumen Height (EAH), First 3 Egg Weight (E3), Early Egg Weight (EEW), Early Production (EPD), Early Shell Quality (EPS), Sexual Maturity (SM), Early Yolk Weight (EYW), Late Albumen Height (LAH), Late Egg Weight (LEW), Late Production (LPD), Late Shell Quality (LPS) and Late Yolk Weight (LYW).

**Figure 2:**
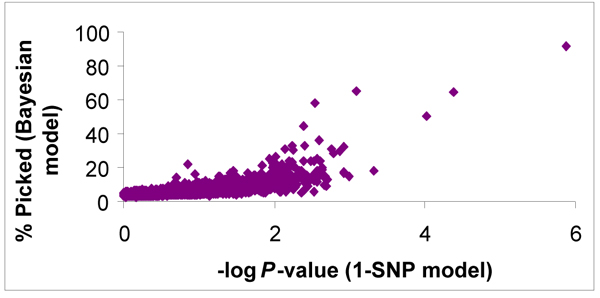
**Comparison SNP associations based on the genome-wise and SNP-wise models**. Plot of the percent of time the SNP was included in the genome-wise Bayesian model vs. -log(*P*-value) from the 1-SNP model analysis for 12 production traits in Line 1.

**Figure 3:**
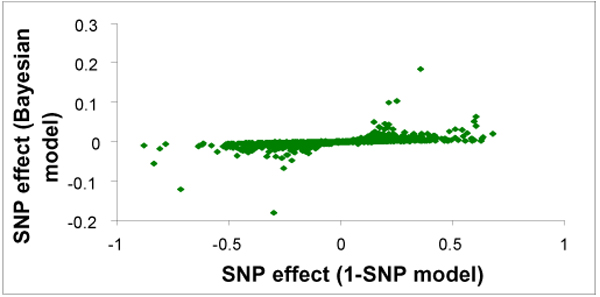
**Comparison of estimates of SNP effects from genome-wise and SNP-wise models**. Plot of estimates of SNP effects from genome-wise Bayesian analyses vs. from 1-SNP analyses for 12 production traits in Line 1. Estimates of SNP effects were standardized by dividing by the estimated genetic standard deviation provided for the traits by Hy-Line, International.

Of the 2730 successfully genotyped SNPs in Line 1, 939 and 802 SNPs had minor allele frequencies (MAF) >0 and ≥ 0.05, respectively. The SNP-wise 1- and 3-SNP analyses (2 Mb average 3-SNP window size) resulted in 159 and 76 tests with *P *<0.01 across the 12 traits. A total of 63 of the 76 significant 3-SNP tests included SNPs that were also significant (*P *<0.01) in the 1-SNP analyses. For the genome-wise BayesB analyses, 33 SNPs were included in the model more than 20 percent of the time (*φ*_*i *_>0.2) across all traits. The SNPs with *φ*_*i *_> 0.2 are tabulated in Additional file 1 and included 4 SNPs for EAH on GGA5, 18 and Z; 3 SNPs for E3 on GGA1 and 4; 5 SNPs for EEW on GGA2, 3, 19, 24, and 27; 3 SNP for EPD on GGA1, 7 and 20; 4 SNPs for SM on GGA1, 4, 5 and 18; 6 SNPs for EYW on GGA1, 4, 11 and 13; 3 SNPs for LAH on GGA3, 5 and 23; 1 SNP for LEW on GGA6; 2 SNPs for LPD on GGA3 and 24; 2 SNPs for LPS on GGA7 and 12; 2 SNPs for LYW on GGA1 and 2. Generally, SNPs that were included in the BayesB model also tended to have a small *P*-value in the 1-SNP analysis (Figure 2). The correlation between *φ*_*i *_from the genome-wise analysis and the -log of the *P*-value from the SNP-wise (1-SNP) analysis was 0.71 for all tested SNPs and 0.83 for SNPs with *φ*_*i *_> 0.2. The same pattern of agreement between the analyses also occurred for estimates of SNP effects from the genome-wise and the SNP-wise analyses (Figure 3). Generally SNPs with large effects estimated by the BayesB method also showed large effects in the 1-SNP analysis, although this relationship was not consistent across all SNPs. The correlation between SNP effect estimates from these two analyses was 0.72 for all tested SNPs and 0.97 for SNPs with *φ*_*i *_> 0.2. Estimates from the BayesB analyses were, however, considerably smaller than those obtained from the 1-SNP analyses.

Summing *φ*_*i *_for a window of 3 adjacent SNPs (excluding windows that were already identified as containing individual SNPs with *φ*_*i *_> 0.2), identified 10 additional regions with a sum of *φ*_*i *_greater than 0.35. "Additional file 1" presents the chromosomal location of the middle SNP for the ten 3-SNP windows with *φ*_*i *_> 0.35, which included two windows for EYW on GGA1 and 25; two overlapping windows for EEW on GGA1; 3 overlapping windows for EPS on GGA7; one window for LAH on GGA19 and 2 overlapping windows for SM on GGA20. Four out of 10 windows with a sum of *φ*_*i *_> 0.35 had P <0.01 based on the SNP-wise 3-SNP analyses.

## Discussion

### Linkage disequilibrium

The LD in the two lines evaluated was high at short distances (r^2^>0.2 at <2 Mb) but declined rapidly with distance. This high LD was expected for commercial layer lines, which exhibit lower heterozygosity than broiler lines. The decline of LD with distance behaved as expected, with the moving average following the predicted LD curve based on the estimate of the historical effective population size. This LD pattern was consistent between lines, years, and the two chromosomes analyzed. A previous report on broiler lines [10] showed similar results of high LD at short distances and the decline of LD followed the same trend, agreeing with the predictions from the Sved [9] equation. If LD is primarily generated by drift, which will be the major force in these populations given their low effective population size, the extent of LD is expected to be a function of linkage distance, rather than physical distance, as demonstrated by Sved [9]. Thus, the LD relationships that were observed here for chromosomes 1 and 4 are expected to be similar for other chromosomes if physical distances are properly converted to linkage distances. A similar decline of LD with linkage distance across chromosomes was also observed by Aerts et al. [27], although they also found some exceptions to this rule, which they attributed to potential effects of selection on LD. Muir et al. [14] reported that their preliminary conclusion, from comparison of the results of chromosomes 1 and 2 with those of chromosomes 26 and 27, was that "LD was higher in macrochromosomes". Their data were generated by typing two separate regions on each chromosome by SNPs with approximately 1 Kb spacing. However, given the summary nature of writing in that review paper, the details of the calculation of the LD and the specific results are not given; thus, it is not known if our methods and theirs were directly comparable. When we, using other data, convert Kb distances to cM using chromosome-specific cM/kb conversions, we did not find a difference in LD between chromosomes (unpublished data), which suggests that the LD by cM distance relationships can be extrapolated from macro- to micro-chromosomes.

The effective population sizes (Ne) that were estimated in these two layer lines were surprisingly small but it should be noted that the standard errors associated with those estimates were large. Estimates of Ne were larger for Line 2 than Line 1 in the current study, which reflects less inbreeding among brown, than white, egg layers [28,29]. Estimates of Ne in these layer lines were substantially smaller than estimates obtained for the broiler lines studied by Andreescu et al. [10], which is consistent with the higher estimates for broiler than layer lines reported by Muir et al. [14] and consistent with the sex-limited nature of the traits selected for in layers. Although low Ne generally correlates with reduced variation, smaller Ne also allows for an increase in the potential benefit of heterosis [30]. Due to the 3-way or 4-way crossbred nature of most commercial poultry, a reduction of Ne in any individual breeding line would not necessarily eliminate beneficial variation in the commercial bird. A cross of two distinct, but low Ne, lines can increase the amount of genetic diversity in the progeny, letting more genes contribute to the phenotype and enhancing performance [31]. The small Ne estimated in the current study suggest that stringent selection on breeding lines may ultimately produce a better commercial product, partly through effective exploitation of heterosis. This emphasizes the importance of testing and selecting upon crossbred performance in the breeding program.

There was a strong correlation within each line of LD between consecutive years. The LD remained high at short distances after one generation (correlations of 0.80 to 0.92 at < 5 Mb), indicating that markers in high LD with a QTL at short distances will retain high LD in progeny, which is essential for MAS. High correlations were observed both for LD measured by r and r^2^. The within-line LD correlations, however, rapidly declined with increasing distance between markers. Heifetz et al. [11] also showed highly correlated LD across generations at short distances and weaker correlations at long distances, although that study utilized microsatellites rather than SNPs, and much more widely spaced markers.

In the current study, 34% of successfully genotyped SNPs were segregating (MAF>0) in Line 1. This level of heterozygosity is consistent with the report by Muir et al. [14] of about 70% loss of genetic diversity in commercial breeding lines. Intense commercial selection programs for production traits may be a primary reason for the decrease in genetic diversity in commercial lines; however, the major part of allele loss occurred prior to modern agricultural practices [14]. Additionally, the SNP panel used in the current study was primarily selected from SNPs that segregate between breeds or lines, rather than within lines, so a low percentage of segregating markers within line is expected. The proportion of segregating SNPs was lower in the two layer lines studied herein than has been reported for broiler populations [10]. Similarly, Muir et al., [14] reported lower heterozygosity (and higher LD) in a white layer line compared to a broiler line. This difference in LD may be attributed to a lower genetic diversity in layers versus broilers [28,29] and the fact that layer selection is on sex-limited traits, which tends to reduce effective population sizes in breeding programs.

The overall findings indicate that LD at short distance was sufficient to detect QTL. Markers in high LD with a QTL at short distances are expected to retain high LD in the following generation and will consequently be effective for MAS.

### Statistical analyses of SNP associations

The results of the current study showed general agreement in identification of associations of markers with traits between use of SNP-wise and genome-wise association analyses. Estimates of SNP effects from the genome-wise analyses were, however, considerably smaller than those obtained from the 1-SNP analyses, primarily because effects were fitted as random and, therefore, 'shrunken'. Estimates of the effect of a particular SNP and its standard deviations (Additional file 1) were calculated across all samples of the Gibbs chain, including samples in which the SNP was not included in the model and, thus, had an estimate of zero. This also means that standard deviations reported in "Additional file 1" should be interpreted with care because they are not for a continuous distribution.

Application of 3-SNP SNP-wise analyses allowed detecting additional SNP trait-associations that were not detected in the 1-SNP analysis. This suggest that both 1- and multi-SNP analyses should be used, as was also recommended by Zhao et al. [13]. Consideration of windows of three SNPs in the genome-wise analyses also resulted in the identification of additional QTL regions. Consideration of such multi-SNP windows will be more important as SNP density increases, and effects of a QTL could be distributed across multiple correlated SNPs that may be genotyped in the QTL region.

Estimates of heritability for the traits varied from 0.05 to 0.84. Most estimates were considerably lower than heritabilities obtained in routine genetic evaluation of this line. The difference is likely due to the fact that the genotyped individuals are a selected sample from the population, as they were selected to be used as sires to breed the next generation.

There was good agreement in estimates of polygenic and residual variances for the 12 studied traits between the ASReml analysis and the Bayesian model without fitting SNP effects. The estimate of polygenic variance decreased, on average, 50% by fitting SNP effects in the genome-wise model compared to the model without fitting SNP effect. This decrease in the polygenic variance might be speculated to be the result of the prior used in the genome-wise model with SNPs, which was set to half the estimate obtained from ASReml. However, using the same prior (the estimate of sire variance obtained from ASReml) in the genome-wise model with and without SNP effects also resulted in a 38% decrease in the estimate of polygenic variance by fitting SNP effects (analyzed only for E3; data not shown). These results indicate that markers explained an important part of polygenic additive variance, however, about half of the genetic variance was not explained by the markers, which suggests the need for developing higher density SNP panels for genotyping.

### SNP association results

Application of one of the first widely available SNP panels for chickens allowed genotyping at a higher marker density than most previous studies and, therefore, revealed many previously undetected QTL regions. Two adjacent SNPs on GGA18 and one SNP in each of GGA5 and GGAZ were significantly associated with EAH. Considering the short distance (~110 kb) between the two adjacent SNPs on GGA18, they are likely associated with the same QTL. These are novel QTL for EAH, in that none of these SNPs coincide with previously reported QTL for similar traits [4,32]. The SNP on GGA5 associated with EAH was also significantly associated with LAH. This is the single instance in the current study for which one SNP was significantly associated with the same trait measured in two different phases of production. The low number of SNP shared between early and late periods of that same trait is unexpected, given the moderate to high correlations over ages in egg production traits [33]. However, the current study suggests the partial genetic independence of most traits at different ages and, therefore, the importance of identifying QTL that differ over time for the same trait. The correlations between early and late traits are generally lower in selected lines, such as the current study, than in unselected lines [33]. The SNPs that were significantly associated with EEW, LEW SM, LPS also did not coincide with previously reported QTL for similar traits [4,32].

There were also many instances of agreement in location between the regions bearing putative QTL in the commercial breeder lines analyzed in the current study and other, independent populations. Three SNPs were significantly associated with E3 on GGA1 and 4 in the current study. The SNP at 8.2 Mb on GGA4 is in the region where significant associations between microsatellite markers and other egg traits (albumen weight at 53 wk and yolk weight at 33 wk) were found in a cross between Rhode Island Red (RIR) and Green-legged Partrigenous (GLP), a native Polish breed [34]. In this region, QTL for egg weight at 55 wk and albumen weight at 33 wk were also detected in a layer × broiler cross [35]. The SNP on GGA4 at 53.7 Mb coincides with a suggestive QTL region for egg weight at 29 wk in a red junglefowl (RJF) × White Leghorn (WL) cross [36]. Thus, these regions of GGA4 appears to harbor QTL related to egg weight, as verified in multiple studies.

Three SNPs in the current study were significantly associated with EPD, on GGA1, 7 and 20. The SNP on GGA1 at 58.6 Mb is located in the region where a suggestive QTL for egg production was detected in a RIR × WL cross [37] and a Cornish × WL cross [38]. This SNP was also significantly associated with EWY, but its effect on these two traits was antagonistic. The SNP on GGA7 at 33.3 Mb is near the suggestive QTL found for egg production at 26–35 wk in a layer × broiler cross [35].

Three overlapping 3-SNP windows were significantly associated with EPS on GGA7. These windows are likely associated with the same QTL in the region where suggestive QTL for egg shell thickness and egg shell strength at 34 wk were detected in a RIR× WL cross [37]. A suggestive QTL for tibia bone mineral density at 8 wk (as percentage of body weight) was also detected in this region in a layer × broiler cross [39]. Thus, this region on GGA7 appears to contain a QTL related to mineral metabolism (of bone and eggshell), which may be a component of all the various phenotypes measured for the QTL in this region.

Six SNPs, on GGA1, 4, 11 and 13, and two 3-SNP windows, on GGA1 and 25, were significantly associated with EYW. The SNP on GGA1 at 24.7 Mb coincides with a QTL region for egg weight at 29 wk in a RJF × WL cross [36]. The SNP on GGA1 at 58.6 Mb, which was significantly associated with EYW, is adjacent to the SNP significantly associated with LYW at 58.8 Mb. These two SNPs might be associated with the same QTL affecting yolk weight at early and late phases of production. The SNP at 70.7 on GGA4 is close to a region where many QTL have been reported for growth and egg-production related traits (including egg weight, albumen weight, eggshell weight and percentage, egg-specific gravity, egg number, short and long length of egg, eggshell colour and bone traits) in different crosses from about 10 independent studies [4,32].

Three SNPs, on GGA3, 5 and 23, and one 3-SNP window, on GGA19, were significantly associated with LAH. The SNP at 77.3 on GGA3 is located in a region where a parent-of-origin effect QTL with paternal expression for egg weight at 41–60 wk was detected in a RIR × WL cross [40]. Note that, in the present study, only the effect of paternal alleles on performance was evaluated.

Two SNPs were significantly associated with LPD, on GGA3 and 24. The SNP on GGA3 at 44.8 Mb coincides with a previously reported suggestive QTL for overall egg production (number of eggs at 16–55 wk) in a layer × broiler cross [35].

Two SNPs were significantly associated with LYW, on GGA1 (previously discussed) and GGA2. The SNP on GGA2 at 28.4 is close to the QTL location for egg weight at 41–60 wk in a RIR × WL cross [40].

Our citing of results from studies using different breeds in the previous, is to support the finding in terms of QTL location. This does not directly imply that SNPs found to be segregating for QTL in one population will work for another population. However, with higher correlations of LD between lines, one can expect that specific SNPs that have significant association with a trait in one line will also be useful in the other line.

## Conclusion

In summary, in the current study, we have demonstrated the preservation of short-distance LD across generations, an essential element of successful MAS. We have shown the general agreement in identification of SNPs associated with phenotype between use of SNP-wise and genome-wise association analyses. We have verified, in an elite layer breeding line, the QTL regions for several previously reported egg-production and egg-quality traits. We have also identified novel putative SNP-trait associations for egg-production and egg-quality traits in this elite layer line.

## Competing interests

The authors declare that they have no competing interests.

## Authors' contributions

BA managed the data, conducted SNP association and ASReml analyses, drafted the manuscript and was involved in development of algorithms for the BayesB and parentage test analyses. ES performed linkage disequilibrium analysis and drafted the LD sections of the manuscript. JA, PS, JEF and NPO managed the experimental birds, and the collection and in-kind contribution of phenotypic and pedigree data and blood samples for genotyping. AH was involved in development of SAS programs for mixed model analysis. DH was involved in development of algorithms for the BayesB analysis. RLF provided advice and programs for the BayesB and parentage test analyses and advice for C++ programming. JCMD and SJL conceived the study and were responsible for its overall design, management, and communications; secured extramural financial support for the project; and edited the manuscript. All authors read and approved the final manuscript.

## Supplementary Material

Additional file 1 SNPs identified to be associated with traits in Line 1. Click here for file
