# Supplementary material for: Extent and consistency of linkage disequilibrium and identification of DNA markers for production and egg quality traits in commercial layer chicken populations
Source: BMC Genomics. 2009 Jul 14;10(Suppl 2):S2. doi: 10.1186/1471-2164-10-S2-S2 (PMC2966334; doi:10.1186/1471-2164-10-S2-S2)
Supplement: Additional file 1 — SNPs identified to be associated with traits in Line 1. [file 1471-2164-10-S2-S2-S1.doc]

## Additional file 1 -SNPs identified to be associated with traits in Line 1

| **Chromosome** | **Mb^1^** | **NCBI^2^ reference SNP ID** | **Trait^3^** | **SNP-wise^4^** | | |  | **Genome-wise^5^** | | |
| --- | --- | --- | --- | --- | --- | --- | --- | --- | --- | --- |
|  |  |  |  | ***P*-value** | **effect** | **SE** |  | **% of times included in model** | **Effect estimate** | **SD of posterior samples** |
| **1-SNP windows** |  |  |  |  |  |  |  |  |  |  |
| 1 | 24.7 | rs14799366 | EYW | 0.0101765 | 0.11 | 0.04 |  | 20.98 | 0.01 | 0.03 |
| 1 | 58.6 | rs13871180 | EPD | 0.0013320 | 0.68 | 0.21 |  | 29.75 | 0.12 | 0.22 |
| 1 | 58.6 | rs13871180 | EYW | 0.0040766 | -0.11 | 0.04 |  | 24.07 | -0.02 | 0.03 |
| 1 | 58.8 | rs14829253 | LYW | 0.0028399 | 0.25 | 0.08 |  | 57.88 | 0.13 | 0.13 |
| 1 | 63.2 | rs14834021 | EYW | 0.0055399 | -0.10 | 0.04 |  | 30.18 | -0.02 | 0.03 |
| 1 | 89.9 | rs13898002 | E3 | 0.0000418 | 0.78 | 0.19 |  | 64.67 | 0.32 | 0.29 |
| 1 | 122.8 | rs15402046 | SM | 0.0029410 | -0.82 | 0.28 |  | 24.18 | -0.11 | 0.24 |
| 2 | 28.4 | rs15077843 | LYW | 0.1398621 | 0.12 | 0.08 |  | 22.09 | 0.03 | 0.06 |
| 2 | 125.6 | rs13730015 | EEW | 0.0145647 | -0.63 | 0.26 |  | 21.36 | -0.08 | 0.19 |
| 3 | 10.5 | rs15264649 | EEW | 0.0023195 | 0.50 | 0.17 |  | 20.04 | 0.05 | 0.13 |
| 3 | 44.8 | rs16261602 | LPD | 0.0063183 | 1.96 | 0.72 |  | 21.81 | 0.21 | 0.50 |
| 3 | 77.3 | rs15390950 | LAH | 0.0099312 | 0.13 | 0.05 |  | 23.02 | 0.02 | 0.04 |
| 4 | 2.4 | rs15474369 | SM | 0.0040945 | 0.85 | 0.29 |  | 44.51 | 0.29 | 0.38 |
| 4 | 8.2 | rs14425231 | E3 | 0.0032255 | -0.53 | 0.18 |  | 24.19 | -0.07 | 0.15 |
| 4 | 53.7 | rs14469988 | E3 | 0.0095214 | 0.48 | 0.19 |  | 26.21 | 0.08 | 0.16 |
| 4 | 70.7 | rs15613782 | EYW | 0.0025125 | -0.15 | 0.05 |  | 36.39 | -0.03 | 0.05 |
| 5 | 5.8 | rs14350738 | EAH | 0.0000947 | -0.15 | 0.04 |  | 50.20 | -0.04 | 0.05 |
| 5 | 5.8 | rs14350738 | LAH | 0.0058237 | -0.17 | 0.06 |  | 23.22 | -0.02 | 0.05 |
| 5 | 14 | rs14516612 | SM | 0.0078361 | -0.90 | 0.34 |  | 22.23 | -0.11 | 0.24 |
| 6 | 25 | rs15799355 | LEW | 0.0016535 | -0.81 | 0.26 |  | 28.30 | -0.12 | 0.24 |
| 7 | 16.9 | rs16589956 | LPS | 0.0017120 | 6.31 | 2.01 |  | 30.92 | 1.05 | 1.89 |
| 7 | 33.3 | rs15882678 | EPD | 0.0072979 | 0.56 | 0.21 |  | 31.01 | 0.12 | 0.21 |
| 11 | 9.8 | rs15613012 | EYW | 0.0008100 | 0.15 | 0.05 |  | 64.89 | 0.07 | 0.06 |
| 12 | 12.5 | rs15656643 | LPS | 0.0024969 | -6.81 | 2.25 |  | 23.92 | -0.80 | 1.76 |
| 13 | 14.1 | rs14066779 | EYW | 0.0039664 | 0.15 | 0.05 |  | 32.96 | 0.03 | 0.05 |
| 18 | 3.4 | rs15817690 | EAH | 0.0056494 | -0.09 | 0.03 |  | 20.43 | -0.01 | 0.02 |
| 18 | 3.5 | rs15817992 | EAH | 0.0056494 | -0.09 | 0.03 |  | 20.92 | -0.01 | 0.02 |
| 18 | 4.7 | rs13507655 | SM | 0.0065887 | 1.09 | 0.40 |  | 20.39 | 0.11 | 0.28 |
| 19 | 1.5 | rs14116385 | EEW | 0.0026804 | -0.73 | 0.24 |  | 25.21 | -0.10 | 0.22 |
| 20 | 4.5 | rs16164433 | EPD | 0.0115186 | -0.51 | 0.20 |  | 24.96 | -0.08 | 0.17 |
| 23 | 1.4 | rs14288801 | LAH | 0.0063621 | -0.14 | 0.05 |  | 23.73 | -0.02 | 0.04 |
| 24 | 5 | rs16198273 | EEW | 0.0106660 | 0.48 | 0.19 |  | 22.52 | 0.07 | 0.15 |
| 24 | 5.9 | rs16199085 | LPD | 0.0011776 | -2.33 | 0.72 |  | 32.43 | -0.39 | 0.69 |
| 27 | 2.8 | rs13620523 | EEW | 0.0057280 | 0.61 | 0.22 |  | 32.98 | 0.14 | 0.24 |
| Z | 21 | rs13677045 | EAH | 0.0000013 | -0.17 | 0.04 |  | 91.88 | -0.10 | 0.05 |
|  |  |  |  |  |  |  |  |  |  |  |
| **3-SNP windows** |  |  |  |  |  |  |  |  |  |  |
| 1 | 11.3 | rs13715018 | EYW | 0.1093956 |  |  |  | 37.34 |  |  |
| 1 | 115.3 | rs15386529 | EEW | 0.0025556 |  |  |  | 41.45 |  |  |
| 1 | 116.5 | rs13771603 | EEW | 0.0015813 |  |  |  | 38.82 |  |  |
| 7 | 25.1 | rs15864122 | EPS | 0.0214092 |  |  |  | 43.85 |  |  |
| 7 | 25.6 | rs14617583 | EPS | 0.0028268 |  |  |  | 46.48 |  |  |
| 7 | 26.2 | rs14618292 | EPS | 0.0009410 |  |  |  | 37.50 |  |  |
| 19 | 1.5 | rs14116385 | LAH | 0.0248542 |  |  |  | 37.80 |  |  |
| 20 | 12 | rs16174658 | SM | 0.0145044 |  |  |  | 36.66 |  |  |
| 20 | 13.1 | rs14280857 | SM | 0.0145044 |  |  |  | 35.58 |  |  |
| 25 | 1.6 | rs14684461 | EYW | 0.0311387 |  |  |  | 36.18 |  |  |

^1^ SNP location in Mb based on May 2006 chicken (build2) genome assembly [41]

^2^ NCBI: National Centre for Biotechnology Information [42]

^3^ Trait abbreviations: Early Albumen Height (EAH), First 3 Egg Weight (E3), Early Egg Weight (EEW), Early Production (EPD), Early Shell Quality (EPS), Sexual Maturity (SM), Early Yolk Weight (EYW), Late Albumen Height (LAH), Late Egg Weight (LEW), Late Production (LPD), Late Shell Quality (LPS) and Late Yolk Weight (LYW).

^4^*P*-value of significance tests for 1-SNP and 3-SNP analyses, SNP allele substitution effect and standard error (SE) for 1-SNP analysis using SNP-wise models. For 3-SNP tests, the reference SNP ID of the middle SNP is presented in the fourth column.

^5^ In 1-SNP section, marginal posterior probability (presented as % included in BayesB model, 100*$\phi_{i}$) of non-zero SNP associations for traits, SNP allele substitution effect and standard deviation (SD) obtained from genome-wise SNP analyses. In 3-SNP section, sum of 100*$\phi_{i}$for a window of 3 adjacent SNP (excluding windows containing SNP with $\phi_{i}$> 0.2). For these 3-SNP windows, the reference SNP ID of the middle SNP is presented in the fourth column. In both SNP-wise and genome-wise analyses, negative or positive effect indicate that the “0” allele (top strand A allele [19]) had, respectively, negative or positive effect on phenotype.
